# Leachability of Heavy Metals from Lightweight Aggregates Made with Sewage Sludge and Municipal Solid Waste Incineration Fly Ash

**DOI:** 10.3390/ijerph120504992

**Published:** 2015-05-07

**Authors:** Na Wei

**Affiliations:** School of Civil Engineering and Architecture, Wuhan Polytechnic University, Wuhan 430023, China; E-Mail: weina31@126.com; Tel.: +86-134-7709-0249; Fax: +86-027-8395-0207

**Keywords:** sewage sludge, municipal solid waste incineration fly ash, heavy metals, sludge lightweight aggregate

## Abstract

Lightweight aggregate (LWA) production with sewage sludge and municipal solid waste incineration (MSWI) fly ash is an effective approach for waste disposal. This study investigated the stability of heavy metals in LWA made from sewage sludge and MSWI fly ash. Leaching tests were conducted to find out the effects of MSWI fly ash/sewage sludge (MSWI FA/SS) ratio, sintering temperature and sintering time. It was found that with the increase of MSWI FA/SS ratio, leaching rates of all heavy metals firstly decreased and then increased, indicating the optimal ratio of MSWI fly ash/sewage sludge was 2:8. With the increase of sintering temperature and sintering time, the heavy metal solidifying efficiencies were strongly enhanced by crystallization and chemical incorporations within the aluminosilicate or silicate frameworks during the sintering process. However, taking cost-savings and lower energy consumption into account, 1100 °C and 8 min were selected as the optimal parameters for LWA sample- containing sludge production. Furthermore, heavy metal leaching concentrations under these optimal LWA production parameters were found to be in the range of China’s regulatory requirements. It is concluded that heavy metals can be properly stabilized in LWA samples containing sludge and cannot be easily released into the environment again to cause secondary pollution.

## 1. Introduction

Increasing generation of hazardous wastes is one of the main environmental problems in most countries in the world [1,2]. Sewage sludge is generated from wastewater treatment plants in cities during the wastewater treatment process. Most sewage sludges contain various heavy metals such as Zn, Cr, Ni, Cu, Pb and Ag originating from different industrial wastewaters. Heavy metals, when present in excess or under the wrong conditions, can produce multiple toxic effects. In China, due to the rapid growth of wastewater output, sewage sludge, as an inevitable by-product of the treatment process of wastewater, is increasing very fast [3,4]. Sludge is often disposed of in open fields because of the shortage of appropriate disposal facilities, resulting in serious problems because of heavy metals then leaching into groundwaters, surface waters and soils. On the other hand, the output of municipal solid waste incineration (MSWI) fly ash has been over 300,000 tons per year in China and is increasing year by year [5,6]. Due to the presence of high amounts of heavy metals its disposal may also pose a significant risk to the environment [7,8]. At present, various methods for removing or stabilizing heavy metals in sewage sludge and MSWI fly ash have been studied to minimize potential risks to human health and the environment. Heavy metal concentrations in sewage sludge can be reduced by chemical extraction [9], bioleaching [10], supercritical water technology [11] and stabilization [12], while melting, solidification/stabilization, acid extraction, vitrification and sintering have all been used to treat MSWI fly ash [13,14,15]. An effective method proposed for decreasing heavy metals leaching from both sludge and MSWI fly ash is thermal stabilization, which can be achieved using high temperature physicochemical reactions. Well known are the investigations in which the use of different types of sludge wastes was proposed for the production of lightweight aggregate (LWA) [16,17,18].

LWA is used for insulation, a loose fills, in lightweight formulated blocks and lightweight concrete in several countries [19,20,21,22]. Currently, clay is widely used to produce LWA and sludge can be used as a partial replacement for clay in the manufacture of LWA. Production of LWA from different types of sludge is considered to be a very satisfactory economic and environmental alternative [16,17,18], since a starting material with no value becomes a product with important industrial application. Meanwhile, MSWI fly ash has also been used to produce LWA for use in lightweight concrete and a range of other applications [23,24,25,26]. Despite the increasing interest in using sludge and MSWI fly ash as a raw material for processing into LWA, only limited details on parameters influencing properties of LWA containing sewage sludge and MSWI fly ash have been reported in the literature. Especially, there have only been few studies investigating the effectiveness of heavy metals stabilization in LWA made from both sewage sludge and MSWI fly ash [27]. In this work, MSWI fly ash was mixed with sewage sludge to form LWA under various operational conditions. This paper presents the results of the leaching tests carried out with these LWA. The main purpose of the present work is to investigate the influence of MSWI fly ash/sewage sludge (MSWI FA/SS) ratio, sintering temperatures and sintering times on the leaching characteristics of LWA samples made with sewage sludge and MSWI fly ash, and to find optimal LWA production parameters.

## 2. Materials and Method

### 2.1. Raw Materials

Sewage sludge was obtained from the Hanxi Wastewater Treatment Plant in Wuhan, China. MSWI fly ash was obtained from the Suzhou Wastes Incineration Plant in Suzhou, China. Sewage sludge, MSWI fly ash and clay were dried to constant weight at 105 °C, then ground and passed through a 0.154 mm sieve. The chemical characteristics of sewage sludge, MSWI fly ash and clay are shown in Table 1. The total concentrations of heavy metals in the sewage sludge and MSWI fly ash were determined by digesting samples in a mixture of HNO_3_ and HCl according to the SW-846 3052 method [28], and then analyzing them according to the SW-846 6010C method [29]. The results are shown in Table 2. The major heavy metals in sludge identified are Zn, Cu, Cr and Pb, and the same are found in MSWI fly ash.

**Table 1:** Chemical composition of dried sewage sludge, MSWI fly ash and clay (dry weight basis).

Materials	SiO_2_	Al_2_O_3_	Fe_2_O_3_	MgO	CaO	K_2_O	Na_2_O
Sewage sludge	21.5	3.8	4.2	1.5	20.7	0.5	0.4
MSWI fly ash	40.1	14.8	1.4	3.1	18.9	4.1	3.1
clay	70.4	15.2	6.5	0.5	0.6	1.2	0.4

**Table 2:** Heavy metal concentrations of the sewage sludge and MSWI fly ash.

Contents of Trace Elements	Zn	Cu	Cr	Pb	Ni	As	Cd
Content of trace elements in sewage sludge/mg·kg^−1^	1052	391	482	151	34	22	8
Content of trace elements in MSWI fly ash/mg·kg^−1^	3157	581	142	1483	51	85	42

### 2.2. Methods

#### 2.2.1. Preparation of LWA

Sewage sludge, MSWI fly ash and clay samples were weighed and then mixed with a mechanical running mixer. In this study, the weight percentage of clay to that of total dried solids (clay + sewage sludge + MSWI fly ash) was 70%. The mixture was then pelletized into pellets with similar 5–10 mm diameters using a pelletizer. The formed samples were dried at 110 °C in a blast roaster for 24 h and then rapidly shifted into an electric tube furnace (SKQ-6 with a maximum temperature of 1700 °C, the sintering temperature and sintering time may vary in different experiments). After the sintering process, the pellets were naturally cooled until they reached room temperature.

#### 2.2.2. Experiments Design

The laboratory experiments in this study were conducted in two phases. The first phase investigated the effect of the mass ratio of MSWI fly ash to sewage sludge on the leaching characteristic of LWA samples containing sludge and determined an optimal mass ratio. Raw pellets were prepared with MSWI fly ash and sewage sludge at a range of mass ratios: 0:10, 1:9, 2:8, 3:7, 4:6. As mentioned above, sewage sludge, MSWI fly ash and clay samples with different ratios were mixed with a mechanical running mixer. After homogenization, a controlled amount of water was added until the mix consistency allowed formation of approximately spherical 5–10 mm diameter pellets. Then the mixtures were taken to a pelletization machine and pellet aggregates of 5–10 mm diameter were made in this pelletization machine. After the preheated treatment, raw pellets were rapidly sintered at the electric tube furnace at 1050 °C and pellets were sintered for 6 min. After the cooling treatment, the leaching characteristic of the sintered pellets were tested. The second phase investigated the sintering temperature and sintering time on the leaching characteristic of LWA sample containing sludge. Raw pellets were prepared with sewage sludge and MSWI fly ash at an optimal ratio of 8:2 according to earlier experiment results. After the preheating treatment, raw pellets were rapidly sintered at the electric tube furnace at 1000, 1050, 1100, 1150 and 1200 °C. Pellets were sintered for 6, 8 and 10 min, respectively, at each sintering temperature. After the cooling treatment, the leaching characteristics of the sintered pellets were also tested.

#### 2.2.3. Heavy Metal Leaching Test

The standard method for determining the leaching toxicity of solid wastes (raw materials and LWA sample containing sludge) by horizontal vibration extraction procedure (HVEP, GB5086.2-1997) [30] was used to evaluate the leaching characteristics of heavy metals from the raw sludge, MSWI fly ash and LWA samples containing sludge. Table 3 shows the HVEP results of heavy metals in raw sludge and MSWI fly ash. As can be seen, the leaching of zinc (Zn) was comparatively higher than that of other heavy metals. Copper (Cu), chromium (Cr) and lead (Pb) contents were the second highest, whereas nickel (Ni), arsenic (As) and cadmium (Cd) contents were relatively lower. Besides, the leaching concentrations of Zn, Cu, Cr and Pb were both found to exceed the China Identification Standard for hazardous wastes (GB5085.3-1996) [31] and Environmental Quality Standards for Surface Water III (GB3838-2002) [32] and were thus classified as hazardous wastes [31]. It is well known that these four heavy metals are particularly harmful and toxic to human beings and the ecological environment. They can accumulate in living organisms, causing various diseases and disorders [33]. Since these four heavy metals are all toxic [33], their release during utilization will have a potentially negative impact on environmental quality, human health, as well as surface- and groundwater resources. Heavy metal stabilization is thus needed before waste utilization.

**Table 3:** Heavy metal concentrations and leaching test results of the sludge.

Leaching Concentrations and STANDARDS	Zn	Cu	Cr	Pb	Ni	As	Cd
Leaching concentration of sewage sludge/mg·L^−1^	85.3	82.4	34.8	9.9	3.1	0.7	0.1
Leaching concentration of MSWI fly ash/mg·L^−1^	134.7	94.8	28.4	38.5	8.7	1.2	0.2
Identification standard for hazardous wastes (GB5085.3-1996)/mg·L^−1^	≤50	≤50	≤10	≤3	≤10	≤1.5	≤0.3
Environmental quality standards for surface water III (GB3838-2002)/mg·L^−1^	≤1	≤1	≤0.05	≤0.05	≤0.02	≤0.05	≤0.05

For LWA samples containing sludge, leaching is a process in which contaminants transfer from a stabilized matrix to liquid medium, so more attention was paid to the main heavy metals (Zn, Cu, Cr and Pb). Leaching rate is often used to evaluate the leachability of waste and it is often defined as a ratio of the leaching content of heavy metal to the total heavy metal content in LWA sample containing sludge, and can be calculated according to following Equation (1):

(1) $$Leaching\;rate(\%)=\frac{Leaching\;content\;of\;heavy\;metal}{Content\;of\;heavy\;metal\;in\;ceramsite}$$

The total contents of heavy metals in the LWA samples containing sludge were also determined by digesting samples in a mixture of HNO_3_ and HCl according to the SW-846 3052 method [28], and then analyzed according to the SW-846 6010C method [29].

#### 2.2.4. Microstructural Analysis and X-Ray Diffraction (XRD) Analysis

Microstructural investigations of LWA containing sludge with different MSWI fly ash content were carried out by scanning electron microscopy (SEM, JSM-5610LV, JEOL, Tokyo, Japan). In addition, the crystalline phases of the sintered LWA containing sludge were identified by X-ray diffractometry (D8 Advance, Bruker, Karlsruhe, Germany).

#### 2.2.5. Quality Tests for LWA Containing Sludge

Loose bulk density, water absorption rate and compressive strength were employed to characterize the quality of the LWA containing sludge sintered under the optimal parameters and they were all determined using an established procedure described by LWA and its test methods (Part 2. Test Methods for LWA, GB/T 17,431.2-1998 [34]).

## 3. Results and Discussion

### 3.1. Effect of MSWI Fly Ash/Sewage Sludge (MSWI FA/SS) Ratio on the Stabilization of Heavy Metals

**Figure 1:**
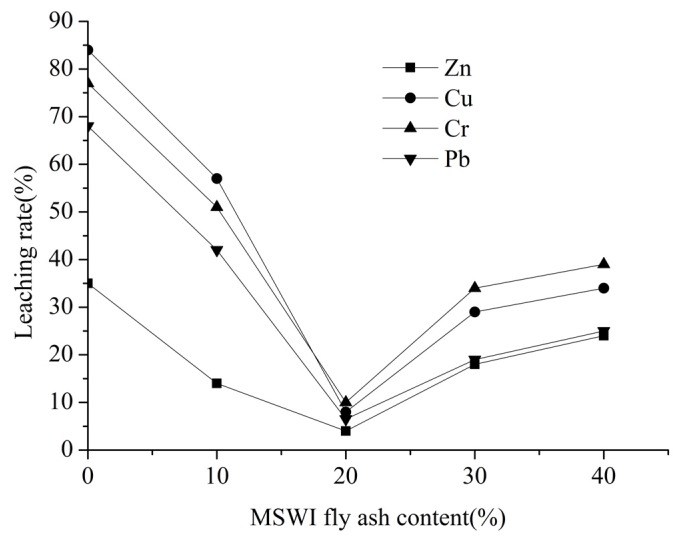
Leaching rate of Zn, Cu, Cr and Pb in LWA containing sludge with influence of MSWI fly ash content.

The leaching results of Phase 1 to examine the impact of MSWI FA/SS ratio on the stabilization of heavy metals (Zn, Cu, Cr and Pb) are shown in Figure 1. Cu showed the highest initial leaching rate and a subsequent significant decrease in the rate as well. With the MSWI fly ash content increasing from 0% to 40%, the leaching rate of Cu decreased from 84% to 8% and then increased to 34%. Similar trends were observed at all other heavy metals. The leaching rate of Cr, Pb and Zn decreased from 77%, 68% and 35% to 10%, 6.5%, 4%, and then increased to 39%, 25%, 24%, respectively. Cr leaching rate exceeded the Cu leaching rate as the MSWI fly ash content above 20%.

The leaching curves for Zn, Cu, Cr and Pb were similar. As can be seen, the leaching rates of all heavy metals (Zn, Cu, Cr and Pb) were highest when the MSWI FA/SS ratio was 0:10 (the MSWI fly ash content was 0%). This may be attributed to the lower acidic oxides (SiO_2_ and Al_2_O_3_) content in sewage sludge than in MSWI fly ash. During the LWA production process, SiO_2_ and Al_2_O_3_, the framework silicate and aluminosilicate compounds that control the viscosity of the material at high temperature, determine the overall crystalline quality and control the crystallization rate. When the MSWI fly ash content is 0%, the SiO_2_ and Al_2_O_3_ in the raw materials were relatively low, and there are insufficient materials to form the LWA skeleton. On the other hand, Gennaro *et al.* [35] also stated that a low SiO_2_/fluxing ratio involves a lower melting temperature and a lower viscosity of the liquid phase, which is unable to entrap a significant amount of gas and thus to bloat during firing. Thus, heavy metals cannot completely solidify within the LWA because of loose structures and semi-developed crystalline phases. The above results demonstrated that the leaching rate of all four heavy metals decreased when the MSWI fly ash content increased from 0% to 20%.

**Figure 2:**
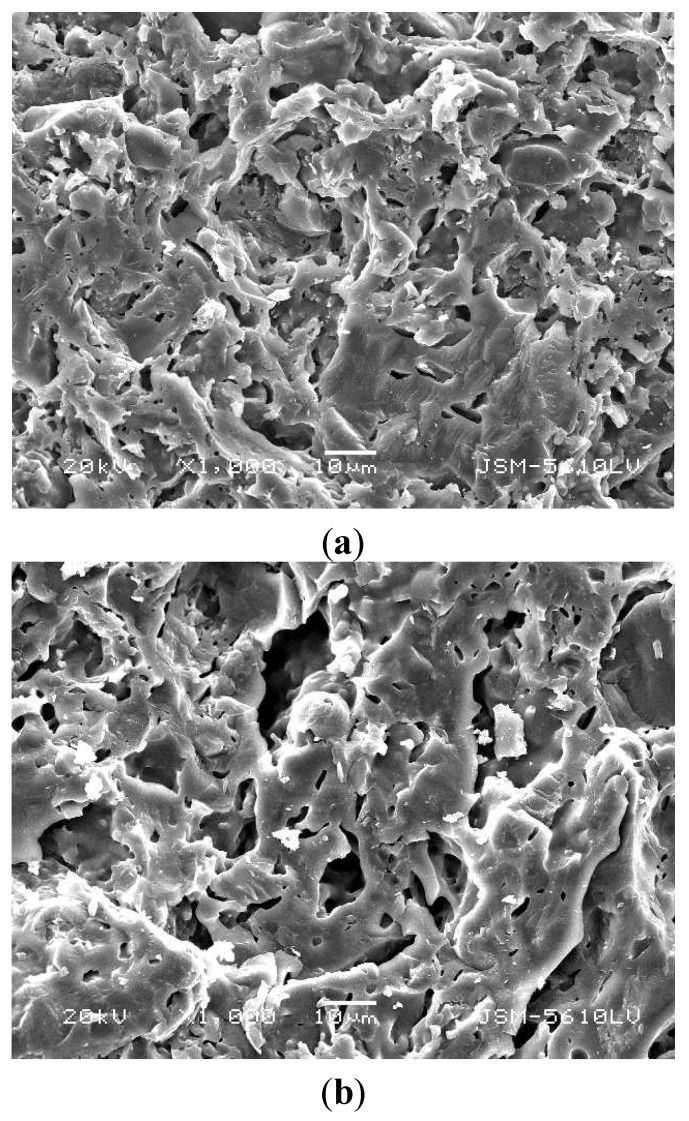
SEM micrographs of LWA containing sludge with different MSWI fly ash contents: (**a**) 20%; (**b**) 40%.

However, the leaching rates of the four heavy metals all substantially increased as MSWI fly ash content increased from 20% to 40%. It was inferred that this variation in the heavy metal leachability was mainly caused by changes in the physical structure of LWA containing sludge. The process of LWA containing sludge production includes three steps: the materials of the raw pellet soften, start melting, and release gases. The gases causing expansion came from the thermally instable materials, such as CO and CO_2_ from the combustion of organic matter, and additional CO_2_ from the dissociation of carbonates. This expansion transformed the pellets into LWA containing a significant glassy phase with evenly-distributed pores. It had been pointed that two conditions are necessary to achieve an appropriate LWA: (a) it should contain sufficient gas producing substances; and (b) on heating, pyroplasticity should occur simultaneously with the formation of gas [36]. Less sewage sludge addition may lead to a reduction in organic matter, which plays an important role in the gas formation and gas expansion. As a consequence, the produced LWA containing sludge has unstable skeletal structures with a large number of cracks and bigger pores. Thus the ability for chemical bonds to form between heavy metals and other components was weak, and more heavy metals were leachable. The effects of MSWI fly ash content on the microstructure of LWA containing sludge are shown in Figure 2. There was a clear evidence of larger pores and more cracks in sintered samples with higher MSWI fly ash content. The SEM observations are in general agreement with the results in Figure 1.

**Figure 3:**
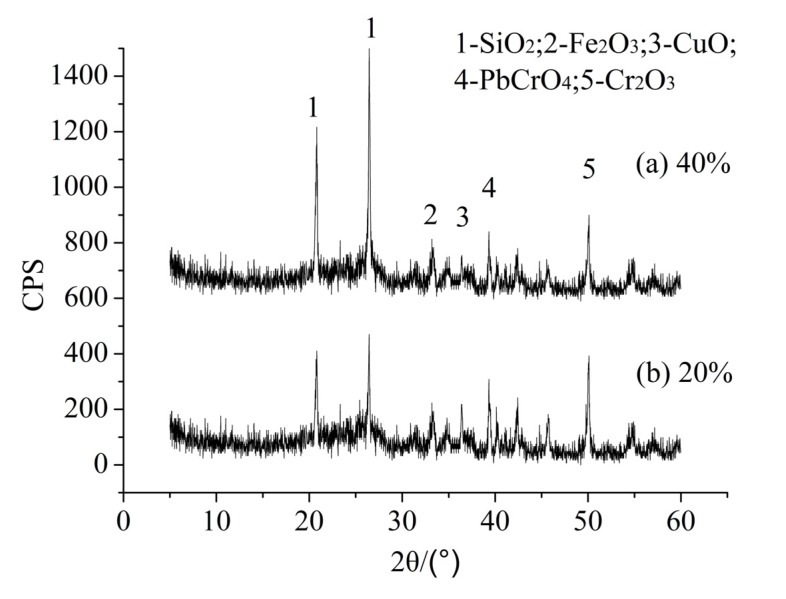
XRD analyses of LWA containing sludge with different MSWI fly ash contents: (**a**) 20%; (**b**) 40%.

Figure 3 gives the XRD data of sintered products with different content of MSWI fly ash. It was shown that the sintered products with 20% and 40% MSWI fly ash exhibited similar X-ray diffraction patterns. The major crystalline phases present were quartz (SiO_2_), hematite (Fe_2_O_3_), copper oxide (CuO), crocoite (PbCrO_4_) and chrome oxide (Cr_2_O_3_). Comparison of XRD data for sintered products with 20% and 40% MSWI fly ash demonstrated that no significant crystallographic changes had occurred. However, there was a significant decrease in the intensity of the quartz peak height as the content of MSWI fly ash decreased, while the intensity of copper oxide (CuO), crocoite (PbCrO_4_) and chrome oxide (Cr_2_O_3_) rose with the content of MSWI fly ash decreasing. The forms of Cr, Cu and Pb in the sintered product suggest that more incorporation of the heavy metals into the aluminosilicates or silicates matrix occurs after the sintering process. It has been reported that the transformation of heavy metals to a crystalline state is advantageous for the long-term stability of the metals and crystalline solids have the improved capacity to bind heavy metals [37]. These results are consistent with the leaching test results seen in Phase 1.

It should be also noted that of the four heavy metals, Zn has the lowest leaching rate and this may attributed to its lower boiling point (907 °C). Due to the low boiling point, large amounts of Zn have been volatilized during the sintering process. Thus both the total concentration and the leaching content of Zn in LWA containing sludge were lower than that of the other three heavy metals.

### 3.2. Effect of Sintering Temperature on the Stabilization of Heavy Metals

**Figure 4:**
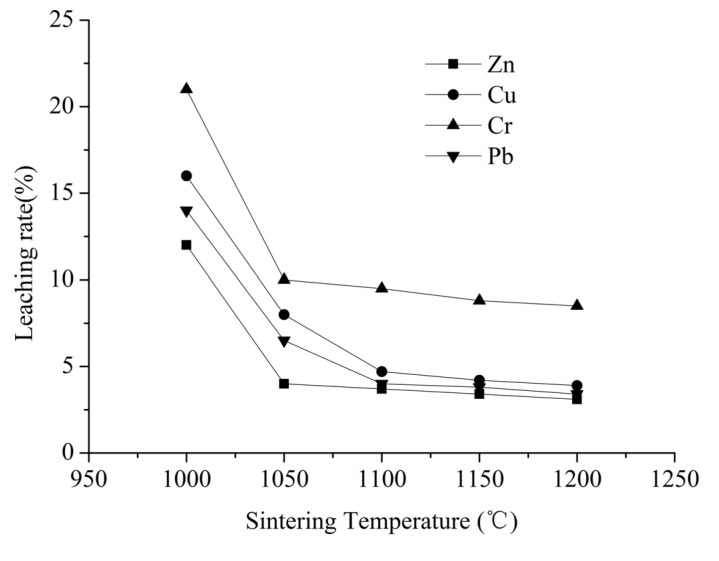
Effect of sintering temperature on the leaching rate of heavy metals.

In the second phase of the experiment, during the sintering process, the pellets were fired at temperatures ranging from 1000 °C to 1200 °C and sintering times ranging from 6 to 10 min. The leaching characteristics of LWA samples containing sludge sintered at different temperature for 6 min are presented in Figure 4. Figure 4 shows the relationship of sintering temperature and the leaching characteristics of heavy metals. When sintered at 1000 °C, Cr leaching (21%) was the highest followed by Cu (16%), Pb (14%) and Zn (12%). With the increasing sintering temperature, the leaching rate of Cr, Cu, Pb and Zn decreased from 21%, 16%, 14%, 12% to 8.5%, 3.9%, 3.4% and 3.1%, respectively. The leaching results of all heavy metals of sintered products demonstrated a descending trend as the sintering temperature increased from 1000 °C to 1200 °C. All the results indicated that the mobility of Zn, Cu, Cr, Pb in LWA samples containing sludge can be reduced by the sintering process. This implies that strong chemical bonds were formed in the LWA samples containing sludge, and the residual amounts of heavy metals in the sintered LWA samples containing sludge were efficiently immobilized within the silicate or aluminosilicate matrix, making heavy metals difficult to leach. Hence, high-temperature sintering can be an effective treatment approach for heavy metals and the higher the temperature, the lower the leachability of hazardous metals. The results are similar to reports of utilizing sludge as a raw material for LWA production [38]. On the other hand, we also observed that the leaching rate of Zn and Cr in the LWA samples containing sludge changed only slightly at temperatures above 1050 °C. Similarly, the leaching rate of Cu and Pb also changed slightly at temperatures above 1100 °C. The results revealed that increasing the sintering temperature above 1100 °C only had a minor influence on the leachability of the heavy metals. Besides, it is shown that all of heavy metals in LWA samples containing sludge prepared above 1100 °C are in compliance with the China Identification Standard for hazardous wastes [31] and the Environmental Quality Standards for Surface Water III (GB3838-2002) [32]. In other words, the toxic metals present in the LWA containing sludge sintered at high temperature (>1100 °C) cannot be easily leached and pose no harmful impact on the environment. So taking cost-saving and lower energy consumption into account, 1100 °C can be considered as the optimal sintering temperature for LWA containing sludge production.

**Figure 5:**
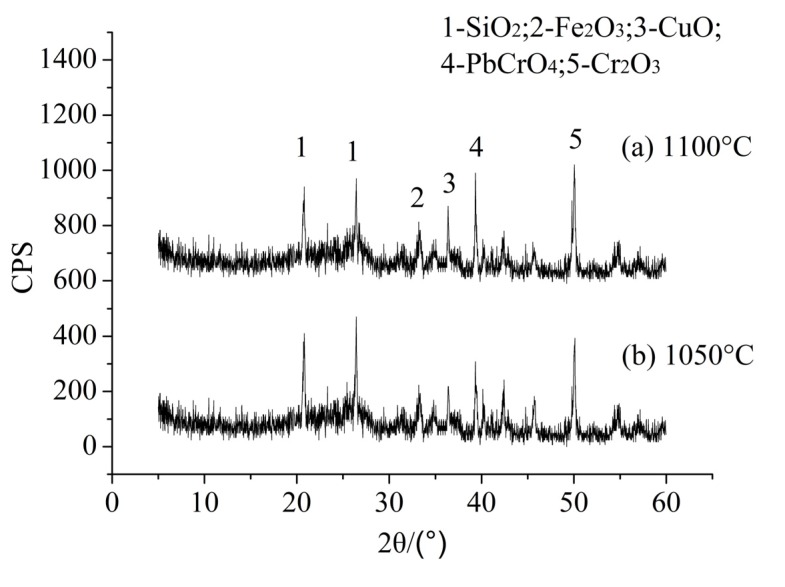
XRD analyses of LWA containing sludge at different sintering temperature: (**a**) 1050 °C; (**b**) 1100 °C.

XRD analyses were also conducted with the sintered products at different sintering temperature. Figure 5 shows that the changes of the sintering temperature have little effect on the formation of the crystals and the major crystalline phases remain almost unchanged. Meanwhile, it was shown that the intensity of copper oxide (CuO), crocoite (PbCrO_4_) and chrome oxide (Cr_2_O_3_) increased with the sintering temperature increase. The results revealed that high temperatures accelerate the formation of CuO, PbCrO_4_ and Cr_2_O_3_, and that heavy metals solidify in the sintered products. These results are also consistent with the leaching test results in phase 2.

### 3.3. Effect of Sintering Time on the Stabilization of Heavy Metals

**Figure 6:**
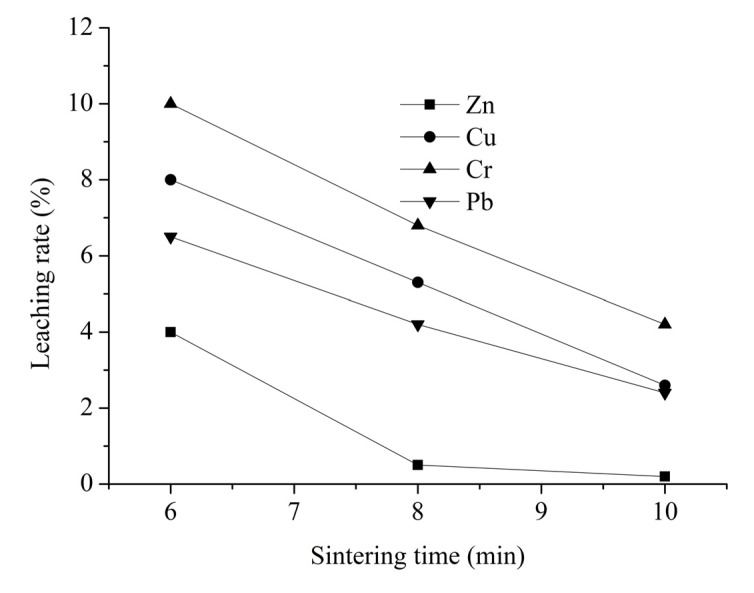
Effect of sintering time on the leaching rate of heavy metals.

The results of Phase 2 to examine the impact of sintering time on the stabilization of heavy metals are presented in Figure 6. Figure 6 shows the leaching characteristics of LWA samples containing sludge sintered at 1050 °C for different sintering times. These leaching rates decreased with increasing sintering time: Cr was initially high (10%) after sintered for 6 min, and reached a minimum of 4.2% after sintered for 10 min; Cu was relatively lower (8%) after sintered for 6 min, and decreased to 2.6% after sintered for 10 min; Pb and Zn decreased more gradually than the above two metals, and decreased to 2.4% and 0.2%, respectively.

As can be seen, leaching rates of four heavy metals all continuously decreased with the increasing sintering time. Thus the prolonged thermal process produces a similar effect in the stabilization of the heavy metals: a decrease in the leaching rate with the increase of sintering time. It can be obtained a conclusion from above results that sintering temperature increase as well as sintering time increase is both advantageous on the stabilization of heavy metals in LWA samples containing sludge. The more effective heavy metal stabilization at higher temperature and longer sintering time has also been reported by other researchers [39]. Besides, it can be seen that Zn is the element most sensitive to sintering time and the leaching rate of Zn in LWA samples containing sludge sintered for 10 min is near 0%.

On the other hand, taking LWA samples containing sludge sintered for 8 min for example, it was shown that, with the exception of Cr, only a small amount of the heavy metals were volatilized during the sintering process, and the leaching rates were all lower than 5%. Additionally, the leaching content of Zn (0.01 mg/L), Cr (0.04 mg/L), Pb (0.03 mg/L), Cu (0.8 mg/L) in this LWA sample (sintered for 8 min) were rather low and meet the maximum content of contaminants for the toxicity characteristic [31,32]. Thus a sintering time of 8 min can be determined as the optimal parameter for LWA samples containing sludge production and the LWA samples prepared can satisfy the nontoxic requirements for the utilization of sewage sludge and MSWI fly ash.

### 3.4. Comparison of Laboratory Produced and Commercial LWA

The LWA sample containing sludge was prepared with the mass ratio of MSWI fly ash to sewage sludge of 2:8, and was sintered at 1100 °C for 8 min. The physical properties (loose bulk density, water absorption rate and compressive strength) of this sintered sample were tested. It was shown that the loose bulk density, water absorption rate and compressive strength of the laboratory produced sample were 0.58 g/cm^3^, 16.5% and 5.4 MPa, respectively and all of the characteristics conformed to the Chinese evaluation standard [40]. Besides, Table 4 shows the varieties of *Arlita*^®^, the highest marketed LWA in Spain, their main features and applications [41]. *Arlita* F5 values shown in this table are the most similar to that obtained from laboratory produced LWA. The value of compressive strength of laboratory produced sample is even higher than that of *Arlita* F5. Thus the laboratory produced LWA may have the similar use to that of *Arlita* F5 and it could be used for concrete slabs, building structures and some other applications. The manufacturing of LWA through the sewage sludge and MSWI fly ash is not only a useful alternative to the extraction of natural aggregates, but also helpful in recycle of the hazardous waste.

**Table 4:** Varieties of *Arlita*^®^, features and applications.

Variety	Loose Bulk Density (g/cm^3^)	Water Absorption (%)	Compressive Strength (Mpa)	Applications
G3	0.325 ± 50	20	0.981	Insulation, Geotechnical Applications, Gardening and Horticulture
F3	0.35 ± 50	20–25	1.962	Prefabricated lightweight strucutures and insulation lightweight concretes
F5	0.55 ± 50	15–20	4.905	Concrete slabs, Building structures
A5	0.575 ± 50	30–35	-	Refractory mortars, Super lightweight concretes

## 4. Conclusions

It can be concluded from the results and discussion above that MSWI fly ash/sewage sludge (MSWI FA/SS) ratio, sintering temperature and sintering time all have a significant influence on the leaching behaviour of heavy metals in LWA samples containing sludge. The leaching rate of Zn, Cu, Cr and Pb all show a significant decrease as the FA/SS ratio increased from 0:10 to 2:8, but then increased again when the FA/SS ratio increased from 2:8 to 4:6. Thus the optimal MSWI FA/SS ratio is determined to be 2:8. Higher sintering temperature and longer sintering time are advantageous for the stabilization of heavy metals. However, the effect is much smaller when sintering temperature and sintering time are more than 1100 °C and 8 min, respectively. Thus it is concluded that the temperature of 1100 °C and sintering time of 8 min are the optimal condition parameters for the production of LWA containing sludge. Besides, the findings of this study reveal that heavy metals in the LWA samples containing sludge prepared under the optimal condition were immobilized quantitatively and would not pose an environmental or human hazard. Furthermore, the loose bulk density, water absorption rate and compressive strength of the optimal-produced LWA samples containing sludge were 0.58 g/cm^3^, 16.5% and 5.4 MPa and they can be used for some applications such as concrete slabs and building structures. This gives evidence that optimal-produced LWA sample containing sludge not only possesses good physical properties, but also poses no harmful effect on the environment.
